# P-1173. NICU MRSA Surveillance: Implications of Retesting Versus Not Retesting MRSA-Colonized Infants

**DOI:** 10.1093/ofid/ofae631.1359

**Published:** 2025-01-29

**Authors:** Julianne Green, Tamika Lovelace, Madelaine Flynn, Elizabeth Palavecino, Werner Bischoff

**Affiliations:** Wake Forest Baptist School of Medicine, Winston-Salem, North Carolina; wake forest Baptist hospital, Winston- Salem, North Carolina; Atrium health, Winston salem, North Carolina; Wake Forest School of Medicine, Winston Salem, North Carolina; Wake Forest University School of Medicine, Winston Salem, NC

## Abstract

**Background:**

Methicillin resistant *Staphylococcus aureus* (MRSA) colonization often precedes invasive infection; decolonization may prevent morbidity and mortality. MRSA surveillance protocols vary widely; at Brenner Children's Hospital (BCH), infants in the neonatal intensive care unit (NICU) undergo repeat testing after decolonization, allowing assessment of MRSA colonization/recolonization events and their relationship to invasive infection.

**Methods:**

MRSA PCR of combined nasal/umbilical swabs from infants in the BCH NICU from 1/1/23 to 2/29/24 were performed on admission and weekly thereafter. Positive infants were decolonized with 5 days of bid intranasal mupirocin and one chlorhexidine gluconate bath (if age-eligible), then underwent repeat weekly testing along with their MRSA-negative counterparts. If/when decolonized infants subsequently tested negative by PCR, no further decolonization occurred unless/until the infant tested positive again (recolonization). Community onset (CO) colonization = a positive MRSA PCR on admission; hospital onset (HO) = at least one negative MRSA PCR prior to a positive.

**Results:**

Of the 1061 infants admitted to the NICU, 93/1061 (9%) were MRSA PCR positive: 16/93 (17%) CO and 77/93 (83% HO), with 2.8 HO colonization events per 1,000 patient days. HO infants took an average of 5 weeks to become newly colonized; 58/77 (75%) were successfully decolonized but 36/58 (62%) of those became recolonized at least once. Long term NICU residents required multiple decolonization cycles- a maximum of 17 cycles for one infant hospitalized for 265 days. Three of four NICU MRSA bacteremia events occurred in HO infants; in each case colonization immediately preceded bacteremia.

**Conclusion:**

Once colonized with MRSA, the majority of neonates underwent repeated decolonization/recolonization cycles. This brings into question the practice of continuous testing after initial decolonization. Events classified as recolonization may actually represent low level carriage in body sites not detected by surveillance, the inability of decolonization to fully eradicate MRSA, or detection of DNA fragments rather than viable organisms.

**Disclosures:**

**All Authors**: No reported disclosures

